# The cost associated with administering risperidone long-acting injections in the Australian community

**DOI:** 10.1186/1472-6963-11-236

**Published:** 2011-09-26

**Authors:** Andrew Dalton, Tim Lambert, Rudolf Schrover, Judy Hertel, Dell Kingsford Smith

**Affiliations:** 1 Centre for Health Policy, Programs and Economics, University of Melbourne, VIC 3052, Australia; 2 University of Sydney, Concord Medical School & Brain and Mind Research Institute, Camperdown, NSW, Australia; 3 Janssen-Cilag Pty Ltd, 1-5 Khartoum Rd, Macquarie Park, NSW 2113, Australia

## Abstract

**Background:**

Risperidone long-acting injection (LAI) is mostly administered twice weekly to people with schizophrenia by nurses at community mental health centres (CMHC) or through mobile outreach visits. This study estimates the cost of resource utilisation associated with the administration of risperidone LAI and the potential savings from substituting two-weekly injections with a longer interval product of therapeutic equivalence.

**Methods:**

A survey of mental health staff overseeing the administration of risperidone LAI at 253 distinct Australian CMHCs was undertaken in November 2009. For the two-week period prior to the survey, respondents were asked questions on injection time (and related tasks) and, for mobile outreach visits, distance and time travelled as well as reduction in visits. Results were stratified by Australian Standard Geographical Classification (ASGC) region. Resource use was quantified and valued in Australian dollars.

**Results:**

Results are derived from 74 CMHCs, representing approximately 26% of the national average risperidone LAI unit two-week sales. Stratified average injection time (including related tasks) for risperidone LAI ranged from 18-29 minutes, with a national average of 20.12 minutes. For mobile outreach visits, average distance per patient ranged from 19.4 to 55.5 km for One Staff Visits and 15.2 to 218.1 km for More Than One Staff Visits, and average time travelled ranged from 34.1 to 54.5 minutes for One Staff Visits and 29.2 to 136.3 minutes for More Than One Staff visits. The upper range consistently reflected greater resource utilisation in rural areas compared to urban areas. If administration of risperidone LAI had not been required, 20% fewer mobile outreach visits would have occurred.

**Conclusions:**

The national average saving per two-weekly risperidone long-acting injection avoided is $75.14. In 2009 in Australia, this would have saved ~$11 million for injection administration costs alone if all patients taking two-weekly risperidone LAI had instead been treated with a therapeutically equivalent long-acting injectable antipsychotic requiring one less injection per month.

## Background

Australia's First National Mental Health Plan promoted the integration of inpatient and community services into a cohesive mental health program [1]. This program includes non-acute patients living in the community receiving outpatient care provided predominantly by local community mental health centres (CMHC) funded through public hospitals [2]. In Australia, there are nearly 6.4 million outpatient service contacts annually involving 327,873 mental health patients at these services [2]. The management of patients with schizophrenia and related disorders accounts for more than one-third of these outpatient mental health service contacts thereby placing a major burden upon mental health care staff [2].

For a significant proportion of community dwelling patients with schizophrenia, maintenance therapy comprises a long-acting injection of an antipsychotic medication administered during these outpatient visits. The therapeutic options for these patients have recently increased with therapeutically equivalent, but longer-acting medication(s) becoming available [3]. A reduced frequency of injections is likely to reduce the workload of mental health care staff, however the need to receive an antipsychotic medication is only one reason why they may need to see a patient with schizophrenia. Thus a reduced frequency of injections will not necessarily correlate one-to-one with any reduction on the frequency of consultations. Any economic evaluation of these new products must therefore consider not only the value of the resource savings per injection no longer required, but also the net impact upon the overall workload of mental health care staff. In Australia, it has been reported that the annual cost of schizophrenia to the public health system is $841 million [4], or approximately $17,000 per patient [4,5]; literature searches though did not identify any published estimates of the resource use and non-medication costs attributable to the administration of an antipsychotic either in Australia or internationally. The Australian Department of Health and Ageing has estimated the national average cost 'per occasion of service' in outpatient psychiatric clinics of public hospitals to be $276 [6], but these visits encompass a broader range of services including general case management and evaluation and do not include allowance for mobile outreach visits.

In administering the injection of antipsychotic medications, CMHCs in Australia organise themselves independently, adopting a variety of approaches that reflect differences in the catchment populations. Patients may receive their injection in their home or area of residence (if homeless) if they are unwilling, or unable, to attend their CMHC for their injection [2]. For the case-management of these patients, including their medication needs, many CMHCs operate a mobile outreach service. When an LAI is required at these outreach visits, case-managers trained in alternative disciplines such as psychology or social work, must be accompanied by a qualified nurse who is eligible to administer injections. The case-manager may also be accompanied by additional colleague/s where a patient presents a security risk to staff. Given the many competing demands upon community mental health staff, the task of administering these injections is onerous, particularly as a significant proportion of case-managers are not qualified to administer injections.

In addition to the cost of the medication itself, resource use for injection administration typically includes labour time of a registered nurse and related tasks such as organising/scheduling an appointment time, obtaining the prescription and medication, reconstituting the injection, and preparing the patient and recording injection details in the patient medical file. Importantly, these activities are required regardless of whether administration of the injection occurs at the CMHC or at a patient's home. Mobile outreach visits involve the additional costs of transport and travel time for all staff attending.

This study estimates the resource use and associated cost of administering an antipsychotic injection, and the impact upon overall costs of the recent introduction of a therapeutically equivalent, longer-acting (monthly), alternative LAI antipsychotic [3]. The subject of the study was risperidone LAI administered two-weekly; the most commonly prescribed long-acting injectable antipsychotic in Australia. The results of this study may also inform the resource burden and potential savings for future funding and allocation of health care resources.

## Methods

We undertook a national survey of mental health professionals responsible for administration of risperidone LAI in the community setting. As it was anticipated that the results of this study would most often be used in economic studies as an estimate of cost-savings when evaluating alternative approaches to clinical practice, a conservative approach to cost-estimation was adopted.

A methodological complexity for this study was an apparent lack of uniformity in appointment scheduling at the CMHC and in the organisation, scale and staffing policies of CMHC mobile outreach services. The most common approach to scheduling appointments is for those case managers at a CMHC who are qualified mental health nurses to devote defined sessions each week purely to administering injections. For the patient, the injection appointment is usually distinct from their case management appointments. Case-managers whose allied health qualifications do not include nursing must arrange a separate appointment time for their clients for a case management session.

A further consideration in the methodological development for this study was the anecdotal evidence that the organisation and commitment of CMHCs to mobile outreach services varied according to the geographic dispersion and diversity of patient population covered by each clinic. For instance, a high proportion of forensic or otherwise unstable patients have implications for the need for additional staff to accompany the mental health nurse administering the injection.

### Survey Development

In order to obtain a broader understanding of the diversity of organisational approaches to administering two-weekly risperidone LAI and to ensure correct identification in the survey of all resources utilised by CMHCs in both metropolitan and regional areas, we consulted with public psychiatrists including CMHC directors (n = 3) and mental health staff located at CMHCs (n = 9). We piloted our questionnaire with four mental health professionals until consensus on format and content was reached. The survey asked the respondent to recall how many patients they had injected with risperidone LAI over the two-weeks prior to completing the survey; the amount of time spent administering risperidone LAI (including related tasks of organising and scheduling the appointment time, obtaining the prescription and medication, reconstituting the risperidone LAI, preparing the patient for injection and recording administration details in the patient's file); whether more than one mental health staff attended mobile outreach visits, as well as the average distance and time travelled; and the frequency of mobile outreach visits.

The survey also sought to quantify the reduction in mobile outreach visits that might arise from administering a lower frequency of injections. Time and travel costs may only be claimed as savings to the extent the number of visits to patients is reduced. Participants were asked how many mobile outreach visits would *still have occurred*, even if an injection of risperidone LAI was *not required*. The framing of the question in this way was considered to be important as a proportion of visits would have still been appropriate for other reasons. That is, when deciding whether a visit would still have occurred, the survey questionnaire explicitly asked participants to consider these reasons by listing examples including the need for counselling or other therapy unrelated to the administration of risperidone LAI. Furthermore, participants were asked to reflect upon the actual cases they had seen over the previous two weeks and provide responses in terms of numbers of visits with and without the need for an injection. Inviting participants to express the effect upon the frequency in this form was thought to be less confounding, and less leading, than asking for results to be expressed as proportional changes in the frequency of visits. Finally, as the purpose of the survey was given as a study of injection administration practice and costs, in order to minimise any bias it did not provide any detail on how the results may be used. The responses to this question enabled estimates of the time and travel savings to be included in the overall estimate of the non-medication savings (i.e. excluding drug costs) from a reduced frequency of injections.

### Survey Administration

**Figure 1:**
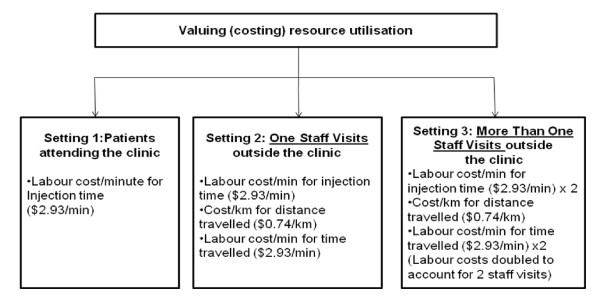
**Valuation of resources from the survey results**.

The authors obtained data on file from Janssen-Cilag Pty Ltd. (the manufacturer of risperidone LAI), to identify all CMHCs in Australia who had placed an order for risperidone LAI. Janssen-Cilag Pty Ltd has developed the database over several years since risperidone LAI was first registered for use in Australia. Surveys were posted to all 253 CMHCs identified in these data. The invitations were thus sent to the entire 'population' of CMHCs known to have administered risperidone LAI at some time, and therefore covered total use of the medication in Australia. The surveys were addressed to either a named responsible mental health staff member(s) where known and having previously consented to receiving survey requests, or to the "team leader" or "centre manager" if specific contact details were not available. However, if more than one survey was returned from one CMHC, such survey(s) were excluded to avoid "double-counting" and potential for over representation of any one CMHC in the survey (Figure 1). Respondents could either mail back their responses or respond online using a "SurveyMonkey™" (http://www.surveymonkey.com/) questionnaire that was otherwise identical to the mailed format. Respondents were offered AUD $100 for completing the survey.

### Survey Analysis

Data from all questions were imported directly into Excel (SurveyMonkey data) or entered via double-entry (paper surveys). Respondents were contacted by telephone to clarify invalid or incomplete responses. During these telephone contacts, no quantitative or other leading information was provided in order to avoid leading or otherwise biasing the responses.

### Resource Valuation

Three patient treatment settings were identified for the purpose of estimating resource use and the costing analysis: Setting 1: Administration of risperidone LAI for patients attending a CMHC; Setting 2: Administration of risperidone LAI at patient visits outside the CMHC (mobile outreach visits) made by one mental health staff; and Setting 3: Administration of risperidone LAI at patient visits outside the CMHC by mobile outreach visits made by two or more mental health staff members.

Resource quantities were calculated for the following variables: (i) labour time: time associated with administering risperidone LAI including related tasks; and (ii) additional resource utilisation for injections administered outside the clinic, as in mobile outreach visits, including travel time and distance travelled, number of staff attending the visit ("One Staff" or "More Than One Staff") and the reduction (%) in the number of visits required if a two-weekly injection of risperidone LAI was no longer required (Figure 1).

The cost of these resources was then estimated. When an injection is required, the main resource to be valued is the time spent by a mental health nurse. The Federal Government's national schedule of fees for medical services appears in the Medical Benefits Schedule (MBS). The MBS lists the approved fees payable for approved health care services by physicians and allied health providers [7], but does not contain an MBS item specifically for an intramuscular injection of an antipsychotic by a mental health nurse. We estimated the cost of labour for administering risperidone LAI based upon our assessment of MBS item numbers for allied health services (refer MBS schedule item numbers 81300 to 81360; available on line [7] which prescribe a fee of $58.85 for a consult of at least 20-minutes for an allied health professional service. Therefore, labour time (cost per minute) was valued at $2.93 per minute derived as $58.85 divided by the national average time for administering an injection of 20.12 minutes as estimated from the survey. As the prescribed fee of $58.85 is for a consult of *at least *20-minutes, the national survey result of 20.12 minutes was used to be consistent with the conservative approach adopted in the methodology. Since the vial preparation, injection and directly related activities for each two-weekly injection of risperidone LAI are the same regardless of whether the administration occurs at the CMHC or outside the CMHC through a mobile outreach visit, the average time for an injection (and cost per minute) is a resource that applies equally to all these patient treatment settings.

The cost per minute of labour time, $2.93 per minute, was also used to value the average time travelled by mental health staff to mobile outreach visits. The cost per kilometre travelled during mobile outreach visits was based on the cost of transport paid as reimbursement to Federal public servants when using private vehicles for official business [8] (the median value of $0.74 per kilometre). For mobile outreach visits involving more than one staff member, it was conservatively assumed that no more than 2 staff attended. For this treatment setting, the injection and travel time costs for both staff were included.

### Cost Analysis

It was thought that organisational practices adopted by CMHCs were likely to differ between rural and urban areas. For example, a greater reliance on mobile outreach programs was likely in areas with a high prevalence of homeless patients, and also in more remote areas. Therefore, we anticipated that an inter-CMHC variation in cost structures would exist. To account for this variation, we identified an independent method of classifying geographic remoteness of each CMHC included in the evaluable data set of survey responses. This was achieved prior to the cost analysis by stratifying survey responses based upon geographical location (postal code) using the Australian Bureau of Statistics (ABS) Australian Standard Geographical Classifications (ASGC) [9]. The ASGC was developed by the ABS for the collection and dissemination of geographically classified statistics, and comprises of six regions: Major Cities of Australia; Inner Regional Australia; Outer Regional Australia; Remote Australia; Very Remote Australia; and Migratory. The last region encompasses offshore, shipping and migratory census collection districts, and was not used in our survey.

**Figure 2:**
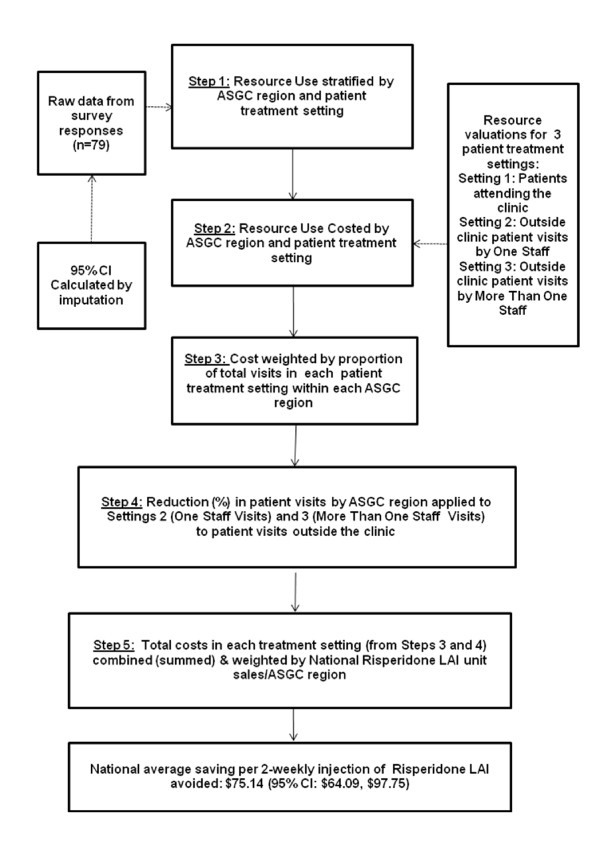
**Overview of the risperidone LAI cost of administration and cost analysis method**.

Costs were calculated by: (i) stratifying by ASGC region and patient treatment setting; (ii) costing the resource use by applying unit costs to attributable labour time and travel estimates; (iii) determining the average cost of resource use when weighted by the proportion of total visits per patient treatment setting within each ASGC region; (iv) calculating the reduction (%) in home visits by ASGC region applied to one staff visits and more than one staff visits; (v) calculating the total costs in each treatment setting by summing steps (iii) and (iv), weighting by the national risperidone LAI unit sales per ASGC region (Figure 2, [10]). We conducted a sensitivity analysis to assess the effect of excluded responses on the primary analysis by including responses from regional Australia that were originally excluded to avoid "double-counting". The data from these responses were included in the evaluable data set and analysed using identical methods.

## Results

### Survey participants

We mailed surveys to 274 health professionals from all 253 identified CMHCs in Australia. Of these 27 (10%) could not be delivered. We received responses from 93 (34%), of which 29 were received online, and 64 by mail. We excluded 14 (15%) of the 93 responses of which 12 (13%) were due to multiple returns occurring from four distinct CMHCs; one (1%) respondent did not provide name or contact details and therefore could not have their responses stratified by AGSC, and another respondent (1%) could not be contacted to clarify an incomplete response. Therefore, 79 of 274 surveys were included in this analysis as evaluable responses - representing a 29% response rate from the known population of CMHCs in Australia. These data are referred to as the "evaluable data set". All respondents comprising the evaluable data set completed every question of the survey.

### Survey representativeness

**Figure 3:**
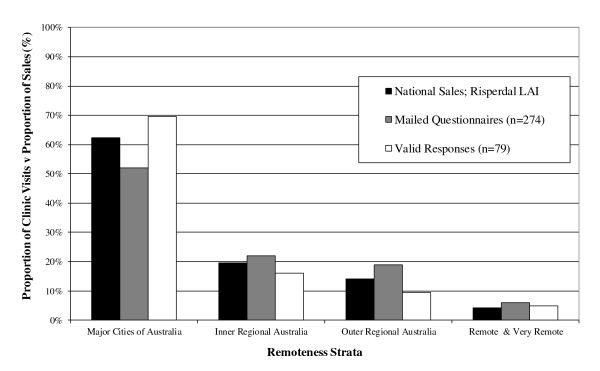
**Comparison of evaluable survey response (n = 79) and national risperidone LAI unit sales by ASGC region**.

Stratification by the evaluable data set by ASGC geographic region revealed a geographic distribution of the 79 responses that was consistent with both the national survey list (n = 274, stratified by ASGC region) as well as that for risperidone LAI national unit sales (2009, stratified by ASGC region; Figure 3, [10]). This analysis confirms the national survey list and evaluable survey population is representative of the community setting in which risperidone LAI is administered nationally.

As only 7 of the 79 responses were stratified to the ASGC regions of 'Remote Australia' (5 responses) and 'Very Remote Australia' (2 responses), these were combined for the presentation of results.

### Data clarification

We successfully clarified 24 invalid or incomplete responses.

### Resource utilisation

The evaluable data set of survey respondents reported information on 3,023 patients visits for the administration of risperidone LAI over a two-week period (Table 1). This equates to the administration of 3,203 units of risperidone LAI, or 26% of the national average risperidone LAI unit sales over a two-week period (based upon IMS national sales data for Risperidone Consta. January - December 2009 [10]). Average injection time, distance and time travelled for mobile outreach visits are shown in Table 2. The upper range of injection time, distance and time travelled reflect a greater resource utilisation in rural areas compared to urban areas. Also included in Table 2 are the respondents' estimates of the reduction in visits that would have occurred if an injection of risperidone LAI had not been required. On average, respondents reported there would have been 20% fewer mobile outreach visits if an injection was not required (Table 3).

**Table 1:** Patient treatment setting stratified by ASGC region

Patient Setting	Major Cities	Inner Regional	Outer Regional	Remote & Very Remote	Total
**Setting 1 **Patients attending clinic	1,524 (72.3%)	346 (71.3%)	221 (77%)	101 (70.1%)	2,192 (72.5%)
**Setting 2 **Patients visited by 1 staff member	389 (18.5%)	99 (20.4%)	48 (16.7%)	28 (19.4%)	564 (18.7%)
**Setting 3 **Patients visited by > 1 staff member	194 (9.2%)	40 (8.2%)	18 (6.3%)	15 (10.4%)	267 (8.8%)

Total	2,107 (100%)	485 (100%)	287 (100%)	144 (100%)	**3,023 (100%)**

Note: one patient equates to the administration of one unit of risperidone LAI

**Table 2:** Summary of national average resource-utilisation per administration of a risperidone long-acting injection

Average Resource use	ASGC region: Major Cities (95% CI)		ASGC region: Inner Regional (95% CI)		ASGC region: Outer Regional (95% CI)		ASGC region: Remote & Very Remote (95% CI)	
Injection time (min)	19.4 (19.1 to 19.7)		18.2 (17.7 to 18.7)		23.4 (21.8 to 25.1)		29.0 (27.6 to 30.4)	
National average injection time (min)					20.12			

**Outreach visits**	**1 Staff Visits**	**> 1 Staff Visits**	**1 Staff Visits**	**> 1 Staff Visits**	**1 Staff Visits**	**> 1 Staff Visits**	**1 Staff Visits**	**> 1 Staff Visits**

Distance travelled/Staff (km)	22.9 (20.1 to 25.6)	19.2 (17.2 to 20.1)	19.4 (18.3 to 20.5)	19.5 (18. 3 to 23.1)	66.3 (43.2 to 89.4)	29.2 (15.5 to 42.9)	55.5 (12.7 to 98.2)	218.1 (12.7 to 331.8)
Time travelled/Staff (min)	44.6 (43.0 to 46.3)	41.4 (38.8 to 44.0)	34.1 (32.6 to 35.7)	33.4 (28.4 to 38.4)	54.4 (40.3 to 68.4)	29.2 (20.0 to 38.4)	54.5 (29.6 to 79.3)	136.3 (70.8 to 201.8)

Reduction in outreach visits (%)	18.0 (0 to 40.3)	19.6 (0 to 49.6)	21.2 (0 to 58.1)	22.5 (0 to 70.9)	41.7 (4.0 to 79.0)	38.9 (0 to 100)	21.4 (0 to 67.6)	13.3 (0 to 100)

**Table 3:** Summary of reduction in mobile outreach visits if an administration of risperidone long-acting injection was not required (over a two-week period)

Average Resource use	ASGC region: Major Cities		ASGC region: Inner Regional		ASGC region: Outer Regional		ASGC region: Remote & Very Remote	
**Mobile Outreach Visits by CMHC staff**	**1 Staff Visits**	**≥ 2 Staff Visits**	**1 Staff Visits**	**≥ 2 Staff Visits**	**1 Staff Visits**	**≥ 2 Staff Visits**	**1 Staff Visits**	**≥ 2 Staff Visits**
Mobile outreach visits (no.)	9.3	4.6	6.6	2.7	3.2	1.2	4.0	2.14
Mobile outreach visits still required if a risperidone LAI administration was not required (no.)	7.6	3.7	5.2	2.1	1.9	0.7	3.1	1.86

Reduction in outreach visits (%)	18.0%	19.6%	21.2%	22.5%	41.7%	38.9%	21.4%	13.3%

National average reduction in mobile outreach visits if a risperidone LAI administration was not required					20%			

### Cost analysis results

The average cost per administration of a two-weekly injection of risperidone LAI by ASGC region and nationally is provided in Table 4. The national average cost per administration of a two-weekly risperidone LAI injection was calculated to be AUD $115.70 (Table 4). Accounting for a 20% reduction in mobile outreach visits should a two-weekly injection not be required (Table 4), the national average saving per two-weekly risperidone LAI injection avoided would be AUD $75.14 (95% CI: $64.09 to $97.75) as shown in Table 5.

**Table 4:** Average cost of two-weekly administration of risperidone LAI (by ASGC region and nationally)

**Total cost pert two-weekly injection of risperidone LAI (Weighted)**^**a **^**by ASGC region**	ASGC region: Major Cities	ASGC region: Inner Regional	ASGC region: Outer Regional	ASGC region: Remote & Very Remote
**Setting 1: Patients attending a clinic**^**b**^	$41.03	$37.89	$52.83	$59.49
**Setting 2: Mobile Outreach Visits (1 staff)**^**b**^	$37.70	$34.16	$46.28	$55.46
**Setting 3: Mobile Outreach Visits (> 1 staff)**^**b**^	$33.76	$26.05	$20.66	$117.59

**Total cost ($) by ASGC region (Cost Setting 1 + Cost Setting 2 + Cost Setting 3)**	$112.49	$98.10	$119.78	$232.53

**National risperidone LAI unit sales weightings by ASGC regions**	62.4%	19.5%	14.0%	4.2%

**National average cost per two-weekly injection**	**$115.70**			

^a ^the average injection time and related tasks associated with the administration of risperidone LAI is the same regardless of patient treatment setting (attendance at a clinic or mental health staff visiting a patient outside the clinic)

^b ^Weighted as the proportion of patient visits within each ASGC region

**Table 5:** Average saving per two-weekly administration of risperidone LAI avoided (by ASGC region and nationally)

Saving per treatment setting and ASGC region	ASGC region: Major Cities	ASGC region: Inner Regional	ASGC region: Outer Regional	ASGC region: Remote & Very Remote
Setting 1: Patients attending a clinic^b^	$41.03	$37.89	$52.83	$59.49
Setting 2: Mobile Outreach Visits (1 staff)	$15.37^a^	$15.79^a^	$25.98^a^	$24.84^a^
Setting 3: Mobile Outreach Visits (> 1 staff)	$15.01^a^	$12.65^a^	$13.30^a^	$30.99^a^

**Total Savings ($) by ASGC region (Savings Setting 1 + Savings Setting 2 + Savings Setting 3)**	**$71.41**	**$66.33**	**$92.10**	**$115.33**

National risperidone LAI unit sales weighted by ASGC region (%)^b^	62.4%	19.5%	14.0%	4.2%
Savings ($) from two-weekly visits avoided (Weighted)	$44.55	$12.91	$12.88	$4.80

**National average saving ($) per two-weekly injection avoided**			**$75.14**	

### Sensitivity Analysis

To assess the effect of responses from regional Australia which were originally excluded to avoid "double-counting", the data from these responses were included in the evaluable data set and analysed using identical methods. The result was consistent with the primary analysis of the evaluable data set, with only a slightly lower estimate of the national average saving per two-weekly injection avoided of $74.54 or $0.60 less than that obtained in the primary result ($75.14).

## Discussion

The aim of our study was to calculate the non-medication cost (i.e. excluding drug cost) of resource utilisation per administration of risperidone LAI, and the average saving per injection avoided that would be realised if switching from a two-weekly injection to a four-weekly therapeutically equivalent long-acting injectable antipsychotic [3]. Whilst estimating the impact upon the frequency of outreach visits was integral to the methods, an implicit assumption is that other components of the care provided to these patients remain constant. The clinical experience of the authors suggests that this is a valid assumption. Therefore, the estimate of $75.14 represents the potential saving per administration of risperidone LAI averted in the community outpatient setting in Australia but can be applied in health systems internationally where the approach to patient management is similar. For instance, re-weighting of results to accommodate different levels of urbanisation can be approximated from Table 1. From Table 2, local valuations can be derived from the application of local unit costs.

We consider our survey to be representative of the risperidone LAI patient population in Australia: the geographic distribution of the responses were similar to both the distribution of CMHCs using risperidone LAI and the geographic distribution of national risperidone LAI unit sales; and the information collected (3,203 visits involving a risperidone long-acting injection) captured approximately 26% of the two-weekly risperidone LAI use in Australia (based upon 2009 unit sales).

Further, we conducted a sensitivity analysis which confirmed that responses excluded from the evaluable data set to avoid double-counting had a negligible impact on the results of the primary analysis of the national average saving per administration of risperidone LAI avoided (i.e. only $0.60 difference).

Our approach also accounted for inter-CMHC variation due to geographic location, ensuring correct representation of rural versus metropolitan settings by stratifying responses for each CMHC based upon the ASGC region. An alternative approach would be to assume that each distinct CMHC responding was a representative sample of all CMHCs administering risperidone LAI in Australia, which would entail combining the data collected from each survey question and assigning a monetary valued without stratification: an approach which does not account for geographical differences.

An important consideration in estimating the costs directly attributable to risperidone LAI administration involved recognition of the fact that mobile outreach visits serve to monitor patient welfare and thus occur for reasons other than merely to administer medication. Therefore we did not assume that when switching from a two-weekly to a therapeutically equivalent four-weekly long-acting injectable antipsychotic that we could simply halve the frequency of visits and therefore the cost. The study accommodated this issue by asking respondents how many of the patients in the two-week period of the survey would still have been seen if they had not required an injection at that visit. From the difference in these responses, the average proportional reduction in visits could be more reliably estimated.

### Limitations

A limitation of our survey was that the results are based upon respondents' recollections. To minimise recall demands, the survey was restricted to a two-week period prior to completion of the survey.

As staff were not aware of how the information was to be used, and objective responses were required, the payment of $100 for providing a completed survey would not have biased results. It did presumably contribute to a high response proportion of 29%; much higher than would normally be expected from a mail-out survey.

The methodological approach for this study was deliberately conservative. This impacts upon three components of the survey. Firstly, it was assumed that no more than two staff would attend visits. During the consultation leading to the development of the survey, it was evident that more than two staff attending would be unusual with minimal impact upon results, yet to capture the small number of such cases in the survey would have complicated the survey design with adverse impacts upon both the quality of responses and overall survey response rate. Secondly, the estimate of the cost per minute of $2.74 for CMHC staff was based upon the average time for an injection of 20.12 minutes to be consistent with the MBS payment being for consultations of at least 20 minutes. Thirdly, and most importantly, it was not possible to quantify all costs possibly related to the use of risperidone LAI specifically. For instance, differences in storage requirements for refrigeration with risperidone LAI that is not a requirement with other long-acting injectable antipsychotic(s) [3], any impacts upon improved adherence, and resources required for following up patients who do not attend their 2-weekly appointments.

Therefore, the estimated cost per injection of risperidone LAI avoided in our study of $75.14 may underestimate the full cost.

In Australia, increased government attention is being given to improving services for people with mental illness and their families and carers through increasing the clinical and health services available in the community, including providing mental health nurses; and by providing an increase in the mental health workforce [11]. Longer-acting injectable antipsychotics would release CMHC resources nationally in support of this strategy.

## Conclusions

Less frequent injections saves resources, including time, travel and reduced mobile outreach visits. The higher cost of administration of a long-acting antipsychotic in rural and remote regions is consistent with the greater reliance upon mobile outreach visits in these settings. The national average saving of AUD$75.14 per injection avoided could potentially equate to a cost saving of ~AUD$11 million per year if all patients taking two-weekly risperidone-long-acting-injection received instead one less injection per month (based upon 2009 estimates).

## Competing interests

Andrew Dalton is an independent health economist who received consultancy fees from Janssen-Cilag Pty Ltd Australia to conduct the survey and costing analysis. Tim Lambert has received speaker, Advisory Board, and/or consulting fees from Janssen-Cilag Pty Ltd, Eli Lilly, Pfizer and Hospira in the last 2 years. Rudolf Schrover, Judy Hertel and Dell Kingsford Smith are employees of Janssen Australia (a pharmaceutical company of Johnson & Johnson) who market Risperdal CONSTA^® ^(risperidone long-acting injection) and Invega Sustenna^® ^(paliperidone palmitate long-acting injection).

## Authors' contributions

All authors participated in the design of the study and assisted with drafting the manuscript. AD collected the data and performed the statistical analysis. All authors read and approved the final manuscript.

## Pre-publication history

The pre-publication history for this paper can be accessed here:

http://www.biomedcentral.com/1472-6963/11/236/prepub
